# Editorial: Pestivirus: Epidemiology, evolution, biology and clinical features

**DOI:** 10.3389/fvets.2022.1025314

**Published:** 2022-10-18

**Authors:** Fernando Viçosa Bauermann, Kerstin Wernike, Matheus Nunes Weber, Simone Silveira

**Affiliations:** ^1^Veterinary Virology Laboratory, Department of Veterinary Pathobiology, College of Veterinary Medicine, Oklahoma State University, Stillwater, OK, United States; ^2^National Reference Laboratory for Bovine Viral Diarrhea/Mucosal Disease and Schmallenberg Virus, Institute of Diagnostic Virology, Friedrich-Loeffler-Institut, Greifswald-Insel Riems, Germany; ^3^Laboratório de Microbiologia, Hospital Veterinário, Instituto de Ciências da Saúde, Universidade Feevale, Novo Hamburgo, Brazil; ^4^Laboratório de Biologia Molecular, Faculdade de Medicina Veterinária, Universidade do Oeste de Santa Catarina (UNOESC), Xanxerê, Brazil

**Keywords:** *Flaviviridae*, APPV, BVDV, CSFV, HoBi-like pestivirus

The genus *Pestivirus* in the family *Flaviviridae* encompasses a wide diversity of ever-evolving and emerging pathogens with a significant economic impact on livestock species, especially ruminants and swine. Among these viruses, bovine viral diarrhea virus (BVDV) types 1 and 2 (officially classified as *Pestivirus A* and *B* species) are endemic in most regions of the world. Conversely, a more recently discovered counterpart, the HoBi-like pestivirus (HoBiPeV, *Pestivirus H* species), appears mainly restricted to South American and Asian cattle populations. Although HoBiPeV and BVDV share many genetic, epidemiological, and clinical features, HoBiPeV is a unique pathogen that may pose a challenge to pestivirus control in cattle populations (1).

As one of the greatest threats to the swine industry worldwide, and despite all efforts toward eradication, the classical swine fever virus (CSFV, *Pestivirus C* species) is still present in specific regions and wildlife populations (2). Understanding the epidemiology of CSFV in wild boars is essential to increase control effectiveness while mitigating dissemination risks to commercial swine populations.

Congenital neurological syndrome is another important swine disease. The etiology, however, is not fully elucidated. Remarkably, the recently identified atypical porcine pestivirus (APPV), classified as *Pestivirus K* species, was described as one of the pathogens involved in the syndrome (3–5). This exciting Research Topic “*Pestivirus: Epidemiology, evolution, biology and clinical features*” sheds light on further understanding of this dynamic group of viruses toward supporting improved control efforts.

The ruminant pestiviruses are the focus of two articles. Barreto et al. reported congenital neurological disease associated with HoBiPeV infection in a newborn dairy calf in Brazil. This report contributes to the understanding of the etiopathogenesis of HoBiPeV infection in cattle since neurological disease associated with HoBiPeV is not often reported. A newborn dairy calf showed neurological signs such as motor incoordination and ataxia, and died in a few days. The main histopathologic findings were predominantly neurological and seemed to affect neurons within several anatomic regions of the central nervous system, resulting in neuronal necrosis at the cerebrum, necrosis, and the degeneration of Purkinje cells of the cerebellum and brain stem, and mild neuronal necrosis at the spinal cord.

Falkenberg et al. conducted a serosurvey for ruminant pestivirus exposure using sera from stray Mexican-origin cattle captured crossing into southern Texas. A virus neutralization assay was used to determine the seroprevalence of BVDV-1, BVDV-2, and HoBiPeV. Approximately 50% of the samples were seropositive for pestiviruses; all the seroreactive samples were positive for BVDV-1, and 49.3% of the samples were positive for BVDV-2 and HoBiPeV. Titers were clearly higher against BVDV-1, and only one sample had a titer clearly higher against BVDV-2. No sample had an antibody titer higher for HoBiPeV than for BVDV. This study provides evidence on the susceptibility of animals that may enter the United States, with ~50% of the animals seronegative for bovine pestiviruses. It also provides evidence that the prevalence of BVDV-1 is more predominant than the other pestiviruses tested.

CSFV was the subject of two articles focusing on in-depth analyses of previous disease outbreaks. Strong et al. applied a molecular epidemiological approach and retrospectively investigated samples collected during a classical swine fever (CSF) outbreak in the UK in 2000. By phylogenetic analysis of full-length CSFV sequences, clusters of closely related viruses could be identified, thereby confirming some of the transmission routes inferred by epidemiological investigations at the time of the outbreak. Other, more distinct viral sequences, however, led to questions about the transmission pathways previously implicated. Hence, monitoring of virus evolution during outbreaks in real-time by using full genome sequencing could be a beneficial tool in addition to traditional epidemiological methods to rapidly identify viral transmission pathways.

To combat outbreaks and to evaluate the effectiveness of countermeasures, it is required to understand disease characteristics such as morbidity and lethality rates among others. To estimate those factors, information about the host population dynamics is required, which is often challenging when wildlife species are affected. Matsuyama et al. estimated the lethality rate, recovery rate, and case fatality ratio of CSF in the wild boar population in Japan, without detailed data on the wild boar population, by using a mathematical model that was constructed to describe the dynamics of the disease and the wild boar population. The authors found a lethality rate similar to estimates in previous studies but a lower recovery rate and suggested that long-term transmission experiments are needed to elucidate the average recovery rate and its variance in wild boar in more detail.

The APPV was the subject of one article, which evaluated complete E2 gene sequences from China. Ma et al. observed four distinct APPV lineages from Chinese sequences. The authors also applied Bayesian analysis to infer the time of the most recent common ancestor (TMRCA), which indicated that APPV emergence dated to 1886 (1837–1924). It suggested that the APPV possibly originated in the Netherlands, a country with developed livestock husbandry. Additionally, APPV was introduced into China where Guangdong was a primary seeding population together with Central and Southwest China as epidemic linkers. APPV is associated with congenital tremors in newborn pigs and has been reported worldwide since its first description in 2015 (6). However, little is known about its origin and dispersion and the work of Ma et al. increases the knowledge about the evolutionary history of APPV in China and worldwide.

The outstanding and exciting work presented in this Research Topic further demonstrates the importance of research on the known pestivirus threats and the pathogenic potential of newly discovered pestiviruses or their association with diseases once deemed idiopathic. The combination of the constant genetic evolution of pestiviruses with the potential of cross-species transmission warrants continuous efforts to restrict the economic consequences of infection in livestock species and support animal welfare and food security.

## Author contributions

FB wrote the introduction. SS, MW, and KW wrote the central part with comments on the cited papers and references. All authors contributed to the article and approved the submitted version.

## Conflict of interest

The authors declare that the research was conducted in the absence of any commercial or financial relationships that could be construed as a potential conflict of interest.

## Publisher's note

All claims expressed in this article are solely those of the authors and do not necessarily represent those of their affiliated organizations, or those of the publisher, the editors and the reviewers. Any product that may be evaluated in this article, or claim that may be made by its manufacturer, is not guaranteed or endorsed by the publisher.
